# Rendu-Osler-Weber disease: a gastroenterologist’s perspective

**DOI:** 10.1186/s13023-019-1107-4

**Published:** 2019-06-07

**Authors:** Annalisa Tortora, Maria Elena Riccioni, Eleonora Gaetani, Veronica Ojetti, Grainne Holleran, Antonio Gasbarrini

**Affiliations:** 1 Fondazione Policlinico Universitario A Gemelli IRCCS, Roma, Italy; 2 Università Cattolica del Sacro Cuore, Roma, Italy; 3 Department of Clinical Medicine, Trinity College Dublin, Dublin 2, Ireland

**Keywords:** Rendu-Osler-weber syndrome, Hereditary hemorrhagic teleangectasia, Gastrointestinal bleeding, Anemia, Gastrointestinal bleeding

## Abstract

Hereditary hemorrhagic teleangectasia (HHT, or Rendu-Osler-Weber disease) is a rare inherited syndrome, characterized by arterio-venous malformations (AVMs or Telangiectasia). The most important and common manifestation is nose bleeds (epistaxis). The telangiectasias (small AVMs) are most evident on the lips, tongue, buccal mucosa, face, chest, and fingers, however; large arterio-venous malformations can also occur in the lungs, liver, pancreas, or brain. Telangiectasias in the upper gastrointestinal tract are known to occur, however data regarding possible small-bowel involvement is limited due to technical difficulties in visualizing the entire gastrointestinal tract. The occurrence of AVMs in the stomach and small bowel can result in chronic bleeding and anaemia. Less frequently, this may occur due to bleeding from oesophageal varices, as patients with HHT can develop hepatic parenchymal AVMs or vascular shunts which cause hepatic cirrhosis and portal hypertension. Gastroenterologists have a crucial role in the management of these patients, however difficulties remain in the detection and management of complications of HHT in the gastrointestinal tract.

## Background

Hereditary hemorrhagic telangiectasia (HHT, or Rendu-Osler-Weber disease) is a rare inherited syndrome, with autosomal dominant transmission, characterized by arterio-venous malformations (AVMs or telangiectasia) which can occur in any organ of the body. Three genetic mutations have been identified: ENG (HHT1); ACVRL1 (HHT2); and more rarely, SMAD4 (found in HHT associated with juvenile polyposis syndrome), however several hundred different mutations have been described. As the genetic mutations in HHT encode proteins responsible for mediating signals from the transforming growth factor-β superfamily in vascular endothelial cells, these genetic defects result in a lack of intervening capillaries and direct connections between arteries and veins [1]. The most important and common manifestation is nose bleeds (epistaxis). In terms of clinical manifestations; telangiectasia (small AVMs) are most evident on the lips, tongue, buccal mucosa, face, chest, and fingers, however; large AVMs can occur in the lungs, liver, pancreas, or brain [2]. The diagnosis is based on the Curaçao criteria: when two of the following criteria are present the diagnosis is “probable” and when three or four criteria are present the diagnosis is “definite”: epistaxis, telangiectasia, visceral vascular malformations, and a first degree relative with HHT. Many patients with diagnosed or undiagnosed HHT, can present with anaemia with no signs of overt bleeding, and in these cases, it is necessary to search for other sites of bleeding, such as the gastrointestinal tract. Other presentations, again in diagnosed or undiagnosed HHT, can occur as a result of hepatic AVMs, with clinical manifestations of portal hypertension, splenomegaly, ascites, and encephalopathy. For these reasons the role of the gastroenterologist is very relevant in the management of patient with HHT.

## Gastrointestinal HHT

Gastrointestinal bleeding is the most common symptom after epistaxis; it occurs in approximately 13–30% of HHT patients, and most commonly begins after 50 years of age [3]. There are many case-reports and case series published in the literature describing patients with overt or obscure gastrointestinal bleeding, however, specific data regarding the occurrence of gastrointestinal bleeding related to HHT is still lacking [4, 5]. In an article by Canzonieri et al. they describe a cohort of 22 HHT patients who underwent gastroduodenoscopy, capsule endoscopy and colonoscopy. The aim of the study was to assess the distribution, number, size, and type of telangiectasia in relation to HHT genotype (ENG or ACVRL1 mutation). They showed that gastrointestinal tract involvement in HHT patients appears to occur more frequently in the duodenum (in 86% of HHT-1 patients and 77% of HHT-2 patients at gastroduodenoscopy; in all HHT-1 patients and in 84% of HHT-2 patients at video capsule endoscopy; and in 1 patient from each group at colonoscopy) [1]. Ha et al. describe a case of HHT-2 with multiple gastric angiodysplastic lesions requiring admission for melena, and stroke due to intracranial hemorrhage, who was treated with conservative treatment (Proton Pump Inhibitors). The patient was hospitalized on a further occasion at which point genetic testing for HHT was performed with the detection of a mutation in the ALK1 gene [6]. Proctor et al. performed enteroscopy in 27 randomly selected HHT patients, and determined that the presence and number of gastric and duodenal telangiectasia correlated with the presence and number of jejunal lesions [7]. A further study by Ingrosso et al. performed gastroscopy and capsule endoscopy in HHT patients and demonstrated a 56% prevalence of small-bowel telangiectasia in patients with HHT [5]. At present, the majority of data reported in the literature is derived from these case reports or case series and to date no large multicentre randomized trials have been undertaken on the management of this special population.

The management of these patients is focused on resolving the acute presentation of anaemia and iron deficiency, and stopping the bleeding or melena, at the site of occurrence in the gastrointestinal tract. Significant problems are related to the need for blood transfusions and a higher morbidity and mortality associated with the anaemia. In these patients, it can often be difficult to diagnosis the disease due to a more advanced age at the onset of symptoms, and due to difficulties in identifying the site of bleeding in the small intestine. The HHT guidelines recommend annual measurement of haemoglobin and serum iron levels beginning at 35 years of age, and suggest that endoscopic evaluation should be performed only in the case of anaemia which is disproportionate to the amount of epistaxis [8]. As is recommended for the management of gastrointestinal bleeding of unknown origin in other contexts, in the case of upper and lower endoscopy negativity, it is recommended to firstly explore the small intestine with capsule endoscopy, and subsequently manage the source of bleeding with deep enteroscopy, if possible [9].

There are many roles of the gastroenterologist in the management of the signs and symptoms of gastrointestinal bleeding in HHT. Firstly, it is important to treat the anaemia. Supportive care includes blood transfusion and iron supplementation (either orally, or intravenously in patients who were inadequately iron-repleted by oral supplementation) [10]. A number of more specific treatments are currently being evaluated; oral estrogen-progesterone or tranexamic acid has been shown to decrease transfusion needs, and a reduction in GI bleeding after IV administration of the anti-angiogenic drug bevacizumab have been reported, however reliable reports of their efficacy in large cohorts or randomized controlled trials are still awaited [11, 12]. The most specific role of the gastroenterologist is to identify the site of bleeding and then, if possible, control it. Argon plasma coagulation (APC) is an effective treatment for the gastrointestinal bleeding, and can be used to AVMs related to HHT. Recent studies have shown the efficacy of APC not only in the treatment of gastric and colonic angiodysplasias, but also in the small intestine, which is the major site of localization of AVMs in HHT patients [13, 14]. A number of case reports have described the use of long-acting somatostatin analogue therapies, such as Lanreotide, in obscure-overt gastrointestinal bleeding, which may be a possible strategy to reduce gastrointestinal bleeding in HHT patients [15].

A further important gastrointestinal problem in HHT patients is related to the SMAD-4 mutation (JPHT). In these cases patients are at risk of developing colonic polyps and subsequently developing colon cancer. The guidelines recommend performing colonoscopy beginning at 15–18 years of age or 5 years younger than at which the youngest family member developed colon cancer, and every 2 years thereafter [16].

## Hepatic HHT

Several case reports regarding the hepatic manifestations of HHT are present in the literature with the estimated prevalence of hepatic involvement ranging from about 74–79% [17–21]. Furthermore, the isolated involvement of the liver is rare. The prevalence of hepatic AVMs depends on the HHT genotype, with a greater frequency of hepatic AVMs in the HHT2 genotype than in the HHT1 genotype [22], and a significant predominance in females (male/female ratio varying from 1:2 to 1:4.5). The hepatic manifestations of HHT include arteriovenous shunts, arterioportal shunts, and portovenous shunts. Large arteriovenous shunts can result in congestive heart failure and the possible development of hepatomegaly and pulmonary hypertension. In these patients, chronic liver ischemia can cause fibrosis and liver cirrhosis. Arterioportal shunts are rare, and are accompanied by arteriovenous shunts. Arterioportal shunts are associated with portal hypertension and its manifestations, such as abdominal ascites or gastrointestinal bleeding due to varices. Other findings on ultrasound or CT scan include a markedly dilated portal vein, hepatic vein, and inferior vena cava with a heterogeneous structure of the liver, nodules and areas of abnormal parenchymal perfusion. In particular, this perfusion abnormality can also compromise hepatocellular regenerative activity leading to focal nodular hyperplasia (FNH), which has a 100-fold greater prevalence in HHT patients than in the general population, or to nodular regenerative hyperplasia [8, 23, 24] Indeed, ultrasound of the liver vasculature shows high velocity and low resistance indices of the hepatic arteries with multiple intraparenchymal hepatic shunts [17, 25]. Other associated manifestations occurring as a consequence of liver involvement include: an increased preload with high-output cardiac failure, portal hypertension, mesenteric ischemia, and biliary disease. In patients with chronic cardiac overload due to liver AVMs, atrial fibrillation has been shown to occur at a rate of 1.6 per 100 person-years, suggesting that caution should be taken in this patient group. Additionally, physicians should be aware of the possibility of developing an anicteric cholestasis due to perfusion anomalies. The main manifestations of biliary disease are biliary stenosis and local cysts or bilomas. In a study describing the occurrence of these anomalies, the authors suggest that the pathophysiology underlying bile duct abnormalities may be related to the increased intra-biliary pressure caused by tortuous ectatic vascularity and biliary ischemia [26, 27]. Encephalopathy, mesenteric angina, or ischemic cholangiopathy with potential hepatic necrosis can also occur but are much rarer [8, 24].

The EASL guidelines recommend screening for hepatic AVMs in asymptomatic individuals with suspected or definite HHT using hepatic Doppler ultrasound as the first line investigation. Second line techniques such as CT or MR scanning are indicated in the differential diagnosis in the case of nodules of the hepatic parenchyma. To determine the hemodynamic impact of liver AVMs on cardiac and pulmonary function it is recommended to perform an echocardiographic evaluation. Patients must be treated with intensive medical therapy in the case of heart failure, portal hypertension, and encephalopathy, and it is important to detect patients that need more intensive treatment at an earlier stage [28]. Buscarini et al. demonstrated that the embolization of hepatic AVMs has led to lethal hepatic infarctions [29]. Orthotopic liver transplantation represents the only definitive curative option for hepatic AVMs in HHT, and is indicated in patients with ischemic biliary necrosis, intractable high output cardiac failure and complicated pulmonary hypertension [8, 24]. Other medical treatments have been proposed, including Bevacizumab, but they have an unpredictable efficacy and non-negligible toxicity [28].

## Conclusions

Hereditary Hemorrhagic Telangiectasia is a systemic disease with multi-organ involvement. The main manifestations are teleangectasias or arterio-venous malformation which can cause bleeding. The most prevalent clinical manifestation is nose bleeding associated with anaemia. Current international guidelines mainly focus attention on the management of anaemia and suggest the use of endoscopy only in patients with severe anaemia which is unrelated to epistaxis [8]. For this indication, the role of the gastroenterologist is crucial in the management of acute and chronic manifestations of HHT. The intestinal and hepatic involvement can negatively affect the progression of the disease and for this reason we believe that in the near future, it will be important to screen all patients for the gastrointestinal manifestations of HHT.

## Data Availability

Not applicable, and for this review all the bibliographic material has been consulted by pubmed.

## Abbreviations

AVMs: Arterio-venous malformations
CE: Capsule endoscopy
DAE: Device assisted enteroscopy
EGD: Esophagogastroduodenoscopy
HHT: Hereditary hemorrhagic teleangectasia
